# Wearable Sensor-Based Exercise Biofeedback for Orthopaedic Rehabilitation: A Mixed Methods User Evaluation of a Prototype System

**DOI:** 10.3390/s19020432

**Published:** 2019-01-21

**Authors:** Rob Argent, Patrick Slevin, Antonio Bevilacqua, Maurice Neligan, Ailish Daly, Brian Caulfield

**Affiliations:** 1 Beacon Hospital, Sandyford, Dublin 18, Ireland; maurice.neligan@beaconhospital.ie (M.N.); ailish.daly@beaconhospital.ie (A.D.); 2 Insight Centre for Data Analytics, University College Dublin, Dublin 4, Ireland; patrick.slevin@insight-centre.org (P.S.); antonio.bevilacqua@insight-centre.org (A.B.); b.caulfield@ucd.ie (B.C.); 3 School of Public Health, Physiotherapy and Sport Science, University College Dublin, Dublin 4, Ireland

**Keywords:** biofeedback, biomedical technology, exercise therapy, orthopedics, mobile health, qualitative, human factors, wearables, inertial measurement unit

## Abstract

The majority of wearable sensor-based biofeedback systems used in exercise rehabilitation lack end-user evaluation as part of the development process. This study sought to evaluate an exemplar sensor-based biofeedback system, investigating the feasibility, usability, perceived impact and user experience of using the platform. Fifteen patients participated in the study having recently undergone knee replacement surgery. Participants were provided with the system for two weeks at home, completing a semi-structured interview alongside the System Usability Scale (SUS) and user version of the Mobile Application Rating Scale (uMARS). The analysis from the SUS (mean = 90.8 [SD = 7.8]) suggests a high degree of usability, supported by qualitative findings. The mean adherence rate was 79% with participants reporting a largely positive user experience, suggesting it offers additional support with the rehabilitation regime. Overall quality from the mean uMARS score was 4.1 out of 5 (SD = 0.39), however a number of bugs and inaccuracies were highlighted along with suggestions for additional features to enhance engagement. This study has shown that patients perceive value in the use of wearable sensor-based biofeedback systems and has highlighted the benefit of user-evaluation during the design process, illustrated the need for real-world accuracy validation, and supports the ongoing development of such systems.

## 1. Introduction

In response to changing global health economics, connected health solutions have the potential to improve the outcomes and accessibility of healthcare [1]. Within rehabilitation, remotely collating and aggregating data from patients has been suggested to have numerous benefits in terms of cost, clinical outcome and patient satisfaction, and can encourage self-management of long-term conditions [2,3]. These connected health solutions can include a biofeedback system which not only gathers data, but also offers the user meaningful information in real-time that is otherwise unavailable to them. This can consist of measurements from the neuromuscular system, or biomechanical variances such as strength or exercise technique [4]. This has led to the development of a number of biofeedback systems utilising a variety of technologies including cameras and wearable sensors [5,6,7,8]. The use of wearable inertial measurement units (IMUs) is one such method of measuring biomechanical variance during exercise and providing feedback to the patient [4,9,10]. The portability of IMUs means that they provide an easy and cost-effective method of capturing human movement data [4,11], and they have been shown to be an accurate method of assessing exercise technique in numerous rehabilitation exercises [11,12,13,14].

IMU based biofeedback systems are particularly suited to the orthopaedic rehabilitation pathway, with an increasing prevalence of surgery and a clearly defined rehabilitation regime. The demand for primary total knee replacement (TKR) for example is estimated to grow 673% in the United States between 2005 to 2030, to almost 3.5 million procedures performed annually [15]. Exercise rehabilitation is the cornerstone of the post-acute recovery process, yet patients report a lack of confidence and vulnerability in the post-operative period [16]. Combine this with the advancement of value-based care, and there is a clear need for interventions to support self-management whilst maximising clinical and cost effectiveness.

Usability is one of the main barriers to widespread uptake of most connected health interventions [17], however there is a distinct lack of both technical and usability validation of sensor-based systems in the peer-reviewed literature [10,18]. To encourage engagement, it is important that the design of biofeedback systems adopts a user-centred iterative process [19], where developers consult end-users to evaluate the system, identify their usability criteria, and understand the perceived benefits and challenges of its implementation in the real-world [20]. To further promote user-engagement, it is also possible to include interventions aiming to increase adherence to exercise within the design of these solutions [21,22].

Thus, this study sought to explore the feasibility, usability, perceived impact, and user experience of an exemplar exercise biofeedback system for orthopaedic rehabilitation in the home. In addition, it was desirable to incorporate user-centred design approaches by encouraging participants to highlight potential refinements or issues in implementation, and to express the criteria they would require in order to maximise engagement and impact.

## 2. Materials and Methods

### 2.1. Participants

A total of 15 patients volunteered to participate in the study (nine females, six males; age: 63 [standard deviation (SD): 8.32]). Participants were recruited from a private hospital in Dublin, Ireland and had recently undergone knee replacement surgery (TKR or unicompartmental knee replacement (UKR)). Participants were required to live within 30 km of the hospital, have no history of cognitive dysfunction, and no difficulty understanding English. The study received ethical approval from the Beacon Hospital Research Ethics Committee (BEA0065), and written informed consent was obtained from all participants prior to commencing the study.

The participants were split into two groups for pragmatic reasons. Group 1 consisted of five participants (Post-Acute) who had all undergone knee replacement surgery at least six weeks previously, and were approaching the end of the acute rehabilitation regime. This group were tested first in order to establish any significant shortcomings in the system that may increase risk of harm to the acute patient group. The second group of 10 participants (Acute) were introduced to the study prior to surgery and then recruited directly from the ward between 2 to 3 days following the operation. This group represents the target user for such a system designed to be implemented in support of discharge home from hospital.

### 2.2. Prototype Exercise Biofeedback System

**Figure 1:**
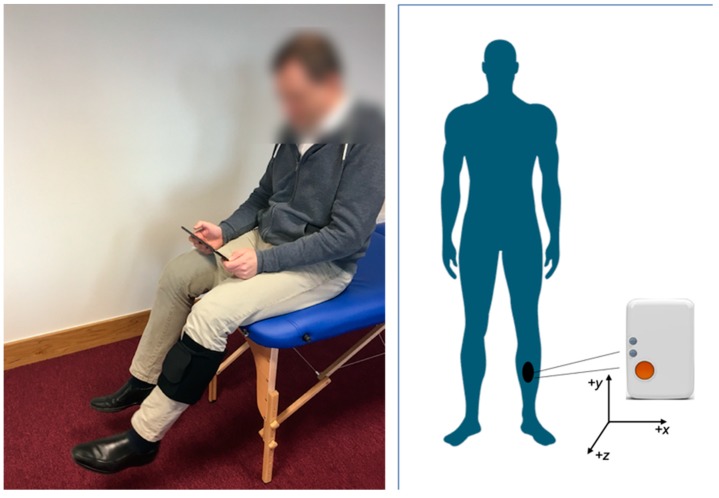
User setup and IMU orientation of the biofeedback system consisting of a single IMU and associated Android tablet application (figure adapted from [23]).

**Figure 2:**
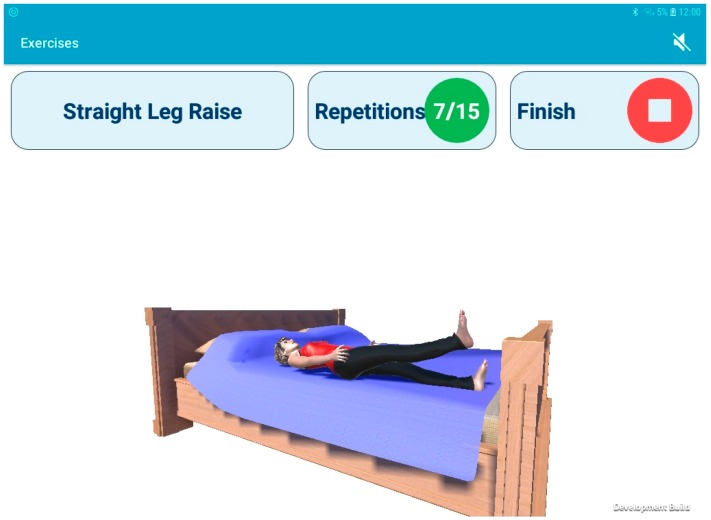
Screenshot of the Android tablet application during the straight leg raise exercise.

The prototype system evaluated by all participants consisted of a single wearable IMU (Shimmer, Dublin, Ireland) [9], and a tablet computer with a custom-built Android application. The Shimmer3 IMU, utilising a tri-axial low-noise accelerometer (±2 g) and tri-axial gyroscope (500 °/s) configured to sample at 102.4 Hz, was placed at the midpoint of the anterior aspect of the shin in a neoprene sleeve, and streamed data via Bluetooth to the tablet whilst the user was guided through their exercises (Figure 1). An avatar mirrored the movements of the user in real-time, the repetitions were counted (Figure 2), and at the end of each exercise, the user was given advice on their technique based on supervised machine learning. 

These methods allow for segmentation and classification of sensor data using support vector machine and random forest techniques described in further detail elsewhere [11,14]. The application also captures patient reported outcomes such as pain and perceived exercise difficulty, and provides all the relevant educational material from the healthcare provider in interactive formats. The user is able to track their progress by viewing their adherence statistics and results of previous patient reported outcome measures such as the Oxford Knee Score or the Western Ontario and McMaster Universities osteoarthritis index [24]. A video of the system can be viewed in the Supplementary Materials (Supplementary File 1).

### 2.3. Experimental Procedure

A mixed methods approach consisting of both quantitative and qualitative data was collected to provide greater insight into the performance of the system, and reduce the weaknesses in using each method in isolation [17,25]. Once signed informed consent was provided, the investigator met participants in their home or at the hospital, whichever was more convenient. Demographic data including age, gender, education, and ownership of mobile technology devices were first collected by self-report. Participants then partook in a user-training session of the biofeedback system lasting approximately 30 minutes, which included completing a full set of the rehabilitation exercises as prescribed in the post-operative protocol. They were then provided with the system and asked to use it at home for the following two weeks to complete the exercises as prescribed by their Physiotherapist.

The investigator met each participant on two additional occasions in their own home during the testing period. During each session, the participant completed a set of the rehabilitation exercises using the biofeedback system, with the investigator observing and making notes on system crashes or user-errors as the participant used the system. After the final session, a semi-structured interview was completed with each participant. Open ended questions were used to establish the perceived impact, usability and user experience of the system, and to explore their opinions on how the prototype could be improved. A Dictaphone was used to record all interview data, and to ensure consistency in questioning, an interview topic guide was constructed based on the aims of the study and the main research questions [26] (Supplementary File 2).

Prior to the final interview, and to provide quantitative data to support the system evaluation, participants also completed two questionnaires; the System Usability Scale (SUS) [27] and the “user version of the Mobile Application Rating Scale” (uMARS) [28]. The SUS is a 10-item questionnaire that has been used to quickly and reliably assess the usability of a system across a number of sectors [27,29]. The output from each user is a score out of 100 which can be used to compare to a growing body of literature to find percentile rankings of a system’s usability performance [30].

The uMARS is designed to be completed by the end-user of mobile applications, rather than the more expert-driven “Mobile Application Ratings Scale” [31]. The application is assessed under the categories of aesthetics, functionality, engagement and information to produce a score out of 5, as well as separate measures to assess the perceived impact of the system and subjective app quality. Similar to previous work [25], this perceived impact section was tailored to identify the perceived impact of the person “exercising with their best technique”.

### 2.4. Data Analysis

All audio data from interviews was transcribed and anonymised. The transcripts were analysed thematically with a grounded theory approach [26]. An early coding template was created based on the interview topic guide which was refined and finalised as themes emerged throughout the analysis [32] by RA (research physiotherapist) and PS (anthropologist). Regular cross-checking was undertaken in a constant-comparison approach ensuring correlation between researchers and reliability of sub-themes [33]. Discrepancies were discussed until agreement was reached, and data saturation was agreed when no further themes and no new data were occurring [26].

The SUS and uMARS scores were calculated following the standard scoring procedure, with the SUS mean and standard deviation (SD) of scores across all participants calculated [27,28,30]. For each participant, a uMARS score out of 5 was calculated under the sections of engagement, functionality, aesthetics and information. The mean of these scores produced an overall score for each participant, perceived impact and subjective app quality were also calculated out of 5 for each user. The means and SDs for the uMARS scores were then calculated across all participants. Estimated adherence rates were also calculated for each user in the acute group. The number of times each participant finished an exercise session was logged within the system and compared with the prescribed number, in order to provide an understanding of the participant’s compliance to the exercise programme.

## 3. Results

All 15 participants completed the semi-structured interview and surveys following two-weeks of using the exemplar biofeedback system. Table 1 illustrates the demographics of participants, including their current access to mobile technology. A summary of the results from interview data are reported in this section, with additional quotations available in Supplementary File 3.

### 3.1. Usability, Functionality and User Experience

The system achieved a mean SUS score of 90.8 (SD 7.8). This places the system above the 95th percentile when compared with published results using this scale [29]. Table 2 displays the results from the uMARS scores, subjective app quality and perceived impact. The results from the functionality section of this survey support this high usability rating with a mean score of 4.2 out of 5 (SD 0.34).

Whilst these scores suggest a high degree of usability for the system evaluated, the qualitative data from interview transcripts provided a greater context and support as to the reason that participants scored the system this way. Specifically, almost all participants commented on how they found the system easy to use, regardless of their perceived literacy with technology:

It’s very simple to use, very simple. And it flows through on a good progression.

*[Acute 2]*

Initially I said to you I wasn’t very computer literate but it’s very simple to use. Once you do it once or twice you can do it with your eyes closed essentially.

*[Acute 9]*

Participants reported positively on their user experience, finding that the system gave them an added incentive to complete their exercises, and provided them with support once they were at home:

I think it set me on a routine very, very quickly and a routine that I actually got to enjoy in a certain way. It was not like holding a sheet of paper… you became involved in it and so user friendly that I really think it was a great aid to me.

*[Acute 5]*

It’s very helpful, it’s much better than leafing through static illustrations. It’s 3D real time. It made what are otherwise boring exercises more interesting.

*[Acute 9]*

Some usability issues were highlighted by participants however, with many reporting inconsistencies in the repetition counter and the technique feedback. Participants also noted that the information presented in the progress section was not displayed intuitively and, on a number of occasions, a bug led to the exercises not being recorded correctly:

There is a bit of problem with the counting in it… Yeah sometimes it misses a few you do… it just runs away with itself.

*[Post-Acute 1]*

Some of the technique feedback seems to be quite inconsistent.

*[Acute 9]*

On the graph I don’t know what the interpretation is supposed to be.

*[Acute 6]*

We had just the one where it says unusual behaviour, unexpected behaviour, please repeat the exercise. I think I was saying to you that on two occasions I actually repeated the exercise… I just said there was a glitch and it didn’t really bother me.

*[Acute 5]*

These issues consequently had a negative impact on user experience. Despite the above participant saying it did not trouble them, other users reported frustration with this, as the exercises are not easy to do and therefore this bug reduced their trust in the system.

It’s just very frustrating as I say it’s on the three that are really painful to do, and you struggle through them and you think well I think I have done them fairly well and then it says unexplained behaviour do them again and you just can’t.

*[Post-Acute 4]*

### 3.2. Perceived Impact

Almost all participants made a reference to the system improving their adherence to the rehabilitation programme, whether that was in the quantity of exercises performed, or the quality with which they completed them:

It kept me doing physio when I might not have done it at home, especially with various things that have been happening at home. So it kept me doing physio and made sure I did it every day.

*[Post-Acute 3]*

Well I can 100% tell you that I had a previous knee operation and I didn’t have an app and I did the exercises as diligently and frequently as I could, but I certainly didn’t do them with the thoroughness and regularity that I’ve done them this time

*[Acute 3]*

One of the main reasons reported for this perceived improvement in adherence largely related to the monitoring provided by such a system provides, be that self-monitoring via the progress graphs or remote monitoring by the clinician:

I found it, I have to say, it made me do the exercises when I didn’t really want to do them, knowing I was being monitored, I do think it helped me a lot.

*[Acute 2]*

I kind of felt that that app now made me do my exercises, the three times a day, and secondly at least it was recording it and you could see, that’s what I liked, you could see the progress from, I’m sure you have the record there of the beginning ones weren’t great.

*[Acute 4]*

Participants reported a motivation for using the system, whether as a result of the monitoring aspect, or from the enjoyment they achieved from using it and improving their self-efficacy:

Oh yes it made a huge difference, that was a great motivation… It just meant that I was in control of my situation and I didn’t feel the days were endless. I was looking forward to doing it to see how well I was doing.

*[Acute 7]*

A huge incentive and even if you try it and say, I’m too tired tonight and then you say no, no I have to do it and I have to try and improve on it.

*[Acute 6]*

Users were keen to point out the importance of the technique classification and felt this was an integral part of the support the system provided:

That’s the important bit about it that it tells you straight away, you need feedback, there is no point in having the app if it doesn’t give you feedback… Because if you are waiting for someone to come in and check it out for you, that’s two to three weeks, but it’s two to three weeks of doing it wrong.

*[Post-Acute 2]*

It’s ideal for somebody that’s coming straight out of hospital and they have to do the exercises on their own, because if you are not doing it correctly why are you bothering doing them in the first place. So that as a tool in itself is worth a hell of a lot.

*[Post-Acute 3]*

Only one participant responded negatively when asked about their overall experience of using such a system. Whilst many others summed up their experience positively, reporting benefits to their confidence and their health literacy regarding the procedure and rehabilitation:

Yes first couple of days that was very interesting and I liked the idea of looking at the little cartoon. But after 2 or 3 days it was for me, I thought it was unnecessary… I have no interest because I know very well if I’m doing better or not. So I find it unnecessary.

*[Acute 1]*

I think the fact that I was almost keen for the next session, it led to a regularity and I think that has paid huge dividends in the exercises and in the result of the exercises on the leg. I really think it was extremely beneficial.

*[Acute 5]*

It was so positive. It was just brilliant. It just meant that I was in control whereas normally I would be coming home and in the hospital it would be altogether different because there’s so much support there, but when you come home its gone except that I had that.

*[Acute 7]*

These comments were supported by the quantitative findings listed in Table 1. Specifically, the high result seen within the perceived impact subsection of the uMARS (4.4/5), which focused on the change in awareness, knowledge and behaviour regarding exercising with the best technique. The subjective app quality scored 4.2/5, with particularly high ratings for when participants were asked if they would recommend the system to others who might benefit (4.6).

### 3.3. Refinements

Participants offered a number of suggestions for refinements or additional features they would like to see to further maximise potential impact in such a system. A large number of participants (*n* = 9) requested that additional exercises were included in the system, including progressions beyond the basic regime:

I think if you gave me progression on the exercises, well done, try this one now, I’d like that.

*[Post-Acute 5]*

Participants also felt that given the specific outcomes and targets following knee replacement surgery, a measurement of their joint angle would be beneficial:

This is probably not possible, but to get the angle of that knee bend, if you knew that…for me that is where I’m really stuck so just to know that…I know it counts it and it said you did it right, but I’m not sure what the angle of the bend is, and I’m rather obsessed with that.

*[Post-Acute 4]*

The other one thing that I’d love to be able to see is you know when you do the knee bends, I’d love to be able to see what angle you got… Now it just you know then the way at least you could say well I’ve gone from a 50 to a 75 rather than just ok it looks like I am doing it ok.

*[Acute 4]*

Other suggestions were based on some of the usability issues discussed previously, with requests for greater clarity in the progress reports, a quality score after each exercise session and improving the graphical interfaces within the system:

And you know the way your Fitbit would have the circle that you have to fill the circle and obviously this system is basic bar charts… Yes that (the Fitbit) does make more sense.

*[Post-Acute 3]*

It would be very difficult to rate it from the previous time, there is no linkage from the previous repetitions… So in a way you don’t know if you are doing better today than you did yesterday… The quality of how I’m doing them.

*[Acute 2]*

I suppose it’s nice to see it really, but she needs to undergo a great makeover… Just a bit more human the graphics… I mean even if it was more a cartoon figure or something it doesn’t really matter.

*[Post-Acute 4]*

Finally, a small number of participants talked about gamification ideas, yet there were mixed opinions on the relevance and the benefit of incorporating gamified features and customisation within the system:

If there was a games element to it you know you have unlocked the next level… or a medal or something.

*[Acute 4]*

I think that’d be a mistake because then you are going to end up turning it into playtime rather than exercise time. I think it’ll lose the point of it. I don’t think you should be able to manipulate too much unless it is information gathering or correcting the programme. For me this is a medical instruction for exercise to improve your health... you’d end up wasting time and not doing the exercise.

*[Acute 9]*

In summary, the SUS and uMARS results, in combination with the interview data, suggest that the system had a high degree of usability and functionality, however there are programming issues causing inaccuracies in feedback that can be improved upon in future iterations to further enhance the user experience of the system. Participants in the acute group demonstrated a mean adherence rate of 79% (range 42%–100%) compared to the recommended number of exercise sessions in their rehabilitation programme. The results suggest that such a system may provide additional support and motivation in the rehabilitation process and improve adherence to the exercise programme, with patients highlighting features such as an extended exercise library and a joint angle measurement tool to further improve engagement and potential impact.

## 4. Discussions

This study investigated patient perceptions of using a custom-built interactive exercise biofeedback system with wearable technology during orthopaedic rehabilitation. Few studies have explored the opinions of the end-user in this population despite the increasing commercial prevalence of such systems [25], therefore this study adds to the current base of literature. Patients largely showed a positive reaction to the use of such a system, with numerous perceived benefits reported, a view that has been shared by clinicians [23]. The results offer fresh insights that can be used to inform the design of such systems, with suggestions to encourage user-engagement, improve usability and optimise impact.

The results indicate that using wearable sensors to offer biofeedback in support of the home-exercise programme prescribed following orthopaedic surgery improves the patient experience and has potential benefits to the clinical outcome. This perceived value may be measured in exercise adherence, patient satisfaction, and clinical effectiveness of the intervention, supporting similar previous suggestions in the literature [20,34], and advocating the ongoing development and evaluation of such systems.

Previous research has demonstrated clinician concern that usability would be a significant barrier to engagement with such systems [23]. However, the results from this study show that, provided the system is built with a user-centred focus, it may be possible to mitigate these concerns, including those of reduced digital literacy within the target demographic. The SUS results reinforce the interview data, as this system scored above the 95th percentile and is comparable to other systems developed for exercise biofeedback [5,25,30]. However few studies have evaluated the usability of a system with a mixed-methods approach over a period of several days as is recommended in the current literature [17].

While usability can be defined as the “effectiveness, efficiency and satisfaction with which specified users achieve specified goals in particular environments”, it is also important to explore the user experience, that is “the users’ perceptions and responses that result from the use of a system” [35]. Results suggest that this system can contribute to an increased sense of routine in the patient’s rehabilitation regime and provide greater levels of enjoyment as they exercise. This in turn may reduce the perceived burden of, and barriers to, self-management [36], thus facilitating better engagement and adherence. However, it also shows how technical issues or wording of feedback can negatively impact user-experience. Particularly given the exercises may be challenging and often painful for patients, they may have a reduced tolerance for technological flaws in a system such as this than they would in other contexts.

It is notable that the uMARS score for the ‘engagement’ section demonstrated distinctly lower mean scores than the other sections. When analysing the individual questions, it is clear that the lack of customisation of sound, content and notifications (mean 1.9) along with the inability to set reminders and allow for interactivity (mean 3.2) had a negative impact on engagement. Arguably though the level of interactivity was misunderstood by participants, as the system will not function without user input from the sensor data. Additionally, an interesting finding from the uMARS was that the aesthetics of the prototype were not of concern, despite the clear graphical issues discussed in the interview data, further highlighting the benefits of the mixed-methods approach.

The usage data would suggest engagement was not as much of an issue as the uMARS would illustrate. The mean adherence rate to the specified number of exercise sessions was relatively high at 79%, with only one participant, who reported negatively on their overall experience in their interview data, demonstrating any kind of drop-off in engagement over time. This patient was one of only three to have received a UKR and was progressed on from these exercises within the study period. These adherence levels are similar to those reported in the recent evaluation of a camera-based biofeedback system for joint replacement rehabilitation [5], and are greater than the varying reports of 35–67% in the wider exercise adherence literature [37,38]. It is also widely documented that there is currently no valid and reliable measurement tool for exercise adherence [38,39]. The use of IMU based biofeedback solutions can therefore not only address factors that affect adherence by providing feedback, goal-setting and self-monitoring, but also offer a more reliable method of measurement of adherence rates [22].

The role of feedback was continuously mentioned throughout this study, with results suggesting it is crucial for habit formation and creating sustained engagement. In current clinical practice there is no ability to monitor or provide feedback to patients between clinic appointments, with a reliance on the patient’s own self-management skills [23]. A system such as this therefore has the ability to offer feedback on exercise technique and clinical progress outside of the clinic setting. This in turn has the potential to provide added incentive to continue engaging with rehabilitation, increasing adherence, and maximising clinical outcomes [40]. Users requested additional features for further feedback such as a joint angle measure, and gamification ideas including unlocking levels of new and more challenging exercises in order to sustain engagement. This highlights the need for user-centred design approaches, as designers cannot assume what the user needs or sees as important [41]. Of particular interest was the role of monitoring and the perceived impact this had, as some patients were motivated by the ability to self-monitor, while others reported greater engagement and adherence as they were aware that their behaviours could be tracked by their clinician.

The lack of real-world validation of exercise biofeedback systems consisting of IMU based sensing platforms in the literature is of concern [10,18]. In this case, users were able to detect what they considered to be inaccurate feedback which has previously been reported as an important criterion for users [42]. These results reinforce the need for field evaluations to become a mainstream methodology, particularly for systems aimed at supporting treatment, where accuracy is key to ensure patient engagement and successful clinical decisions [10].

This study is not without its limitations however, particularly as the sample was selected from a single private hospital. This therefore may not be representative of the wider population, as the majority of patients were degree educated and from more affluent socio-economic backgrounds, which are determinants of better self-management and health outcomes [43,44]. While a sample size of 15 participants is standard for usability testing, this does not guarantee the opinions discussed are generalisable beyond this population. It is important to note that the impact discussed in this study is based solely on participant perceptions from analysis of interview data and uMARS results, and further objective investigation of impact is recommended. As the aim of this paper was to explore user perceptions on usability and perceived impact, there is no reference to the quantitative performance and accuracy of the system in measuring patient exercise technique in the ‘real-world’. Therefore, until this validation is completed, the adherence rates stated could only be calculated by the number of exercise sessions, rather than the exact number of repetitions completed during each session.

Despite these limitations, patients believe exercise biofeedback systems consisting of IMUs and mobile technology can offer significant value to the rehabilitation experience, potentially maximising adherence, satisfaction, and therefore clinical outcome. Few other studies have been published that investigate these perceptions in similar systems, despite the importance of a user-centred focus in the design process. Further research is required to objectively assess whether these perceived benefits are demonstrated in clinical practice, and to rigorously validate the technical aspects of any such system. The development of patient recommended features can also be undertaken to improve the impact and user-experience in this biofeedback system, including a range of motion measurement, additional exercises for progression and gamification elements.

## 5. Conclusions

The emergence of ubiquitous mobile technologies and wearable sensors offer the opportunity to provide novel and effective methods of supporting patients in exercise rehabilitation. Patients perceive the use of wearable exercise biofeedback systems such as the prototype evaluated can offer additional motivation and feedback to enhance adherence, and positively impact patient experience and clinical outcome. However, there is a need for such systems to demonstrate real-world accuracy validation. By involving patients in the development of such systems in a user-centred design manner, it is possible to maximise engagement and effectiveness, and highlight shortcomings or areas for further research early in the development cycle. The prototype system can be considered highly usable and the findings support the ongoing development and evaluation of such sensor-based biofeedback systems.

## Figures and Tables

**Table 1:** Participant demographics and technology ownership.

Demographic Details	*n* = 15	
Marital Status	Married	86.6%
	Single	0%
	Widowed	6.6%
	Other	6.6%
Lives with	Spouse	46.6%
	Family	40%
	Alone	13.3%
Education	Degree Educated	73.3%
	Completed Secondary	20%
	Completed Primary	6.6%
Technology Ownership	Mobile Phone	100%
	Smart Phone	86.6%
	Tablet	66.6%
	WiFi	93.3%
	Health/Fitness App	26.6%

**Table 2:** Results from the user version of the Mobile Application Rating Scale (uMARS). Overall uMARS quality score shown in bold.

uMARS Section (score out of five)	*Mean (SD)*
Engagement	3.5 (0.69)
Functionality	4.2 (0.34)
Aesthetics	4.2 (0.45)
Information	4.4 (0.34)
**Overall Quality**	**4.1 (0.39)**
Perceived Impact	4.4 (0.83)
Subjective App Quality	4.2 (0.86)

## Supplementary Materials

The following are available online at http://www.mdpi.com/1424-8220/19/2/432/s1, Supplementary File 1: Video of system usage, Supplementary File 2: Interview topic guide, Supplementary File 3: Additional quotations from interviews.

## Abbreviations

IMU	inertial measurement unit
SUS	system usability
SD	standard deviation
TKR	total knee replacement
UKR	unicompartmental knee replacement
uMARS	user version of the Mobile Application Rating Scale
