# Supplementary material for: Wearable Sensor-Based Exercise Biofeedback for Orthopaedic Rehabilitation: A Mixed Methods User Evaluation of a Prototype System
Source: Sensors (Basel). 2019 Jan 21;19(2):432. doi: 10.3390/s19020432 (PMC6359655; doi:10.3390/s19020432)
Supplement: Supplementary file 1 [file sensors-19-00432-s001.zip › Supplemental Files/Sensors Supp File 2.docx]

SUPPLEMENTARY FILE 1: INTERVIEW TOPIC GUIDE

**Main questions formed the core structure of the interview, further follow-up questions were put forward dependent on initial response.**

- How do you think using this platform has affected your rehabilitation?

*Example of follow-up questions:*

- *You say you think it has been beneficial, in what way?*
- *Why do you think that?*
- *How did it make you feel when doing that?*
- How did you find the system to use?

*Example of follow-up questions:*

- *What have you explored within the software?*
- *Describe how your skills in using the platform have changed from when you first received it to now?*
- *How did you feel when that happened?*
- *How confident would you feel in using this software for a further period of time independently?*
- What did you think about the way the information was presented in the app?

*Example of follow-up questions:*

- *Tell me what you understood the progress graphs to show you?*
- *What aspect of the presentation could be improved?*
- Did you encounter any difficulties whilst using the software, any tasks that you found difficult or technical issues?

*Example of follow-up questions:*

- *How did you feel when that happened?*
- *Do you remember what you were doing when that occurred?*
- Is there any way this system can be improved based on your experience?

*Example of follow-up questions:*

- *Are there any other features you would like to see?*
- *You say the sleeve isn’t easy to fit, what might make it easier?*
- Do you have any other thoughts about this system, or about using technology in healthcare?
