# Supplementary material for: Wearable Sensor-Based Exercise Biofeedback for Orthopaedic Rehabilitation: A Mixed Methods User Evaluation of a Prototype System
Source: Sensors (Basel). 2019 Jan 21;19(2):432. doi: 10.3390/s19020432 (PMC6359655; doi:10.3390/s19020432)
Supplement: Supplementary file 1 [file sensors-19-00432-s001.zip › Supplemental Files/Sensors Supp File 3.docx]

SUPPLEMENTARY FILE 2

PATIENT INTERVIEW DATA

PRIMARY THEME: USABILITY

| Sub-theme | Description | Quotations | Participant |
| --- | --- | --- | --- |
| Usability Difficulty | References relating to the ease / lack of ease participants reported in using the system. | *Very good, I thought it was very easy to use.* | Post-Acute 3 |
|  |  | *I was worried that trying to learn would take up what should be the learning time for the exercise. So once you went to it once, it was just brilliant after that. And I didn’t have any problem and the thing worked and at one stage it said something the power was low and I knew straight away well just plug it in. And it just came…it’s such an easy transition from knowing nothing to knowing a whole lot of things.* | Acute 7 |
|  |  | *I found it very easy to adapt to it. For what I used it for to do the exercise and record the exercise on it, it was very, very easy.* | Acute 5 |
|  |  | *It was very simple… Yeah it was. And it was effective .* | Post-Acute 4 |
|  |  | *Initially I said to you I wasn't very computer literate but it's very simple to use. Once you do it once or twice you can do it with your eyes closed essentially.* | Acute 9 |
|  |  | *It’s very simple to use, very simple. And it flows through on a good progression.* | Acute 2 |
|  |  | *I found it very easy to get using it and didn’t have any problem with it, I suppose once I used it a couple of times I was quite confident with it.* | Post-Acute 1 |
|  |  | *I never really had any issues with actually using the whole thing… I mean I don’t think I had any difficulty using it from the start so it was just it was plug and play kind of, you know?* | Acute 4 |
|  |  | *the app was very well made and easy to use* | Acute 3 |
|  |  | *Using that is so easy. You just follow the instructions.* | Acute 6 |
| Repetition Counter | References relating to the repetition counter in the exercise component of the system. | *There is a bit of problem with the counting in it… Yea sometimes it misses a few you do… it just runs away with itself.* | Post-Acute 1 |
|  |  | *You knew if the person was moving you were doing it, the only thing that might get to you on occasion if the numbers don’t come up, if you are doing 3 repetitions and it’s only saying one, you are saying hold on I did count 3.* | Post-Acute 2 |
|  |  | *The repetitions are not showing up on some they show up and on some they don’t show up. So I just count that when I have done 15… Sometimes it stays at zero and I just do 15 and then it tells me you did these exercises correctly well done.* | Post-Acute 3 |
|  |  | *The knee bend one unless you get to a certain angle it doesn’t count it, and then occasionally it will double count it will count as you are going up and down.* | Post-Acute 4 |
|  |  | *The repetitions are sometimes a bit skew ways, there has been times when it’s counted twice on the way up and on the way down. And then there is other times it doesn’t record at all, it’s nil out of 15, but when you are finished it says 15…detected or whatever.* | Acute 3 |
|  |  | *The counter’s not working basically… Usually, the minute I lie down and put the thing, it registers one straight away. It might register a second it gives like two freebies kind of thing so keep.* | Acute 4 |
|  |  | *The count doesn’t tend to happen when I'm doing the slide. It does give me a correct mark at the end of it. But it doesn’t tend to count that, equally with my leg lift, it doesn’t tend to count.* | Acute 6 |
|  |  | *The sensor seems to be very sensitive and will record 4 when I've only done 1…. So I count manually to make sure I've done 15.* | Acute 9 |
| Classification Accuracy | References relating to the technique feedback provided by the system. | *Some of the technique feedback seems to be quite inconsistent.* | Acute 9 |
|  |  | *The right or wrong, a lot of the time I knew I was doing it right, and I wasn’t lifting my leg and I wasn’t…yet it might have told me that sometimes I was lifting my leg.* | Post-Acute 3 |
|  |  | *There is another exercise which is I don’t know how it scores… Yes the towel, so you lift too high and no I didn’t.* | Acute 1 |
|  |  | *I think no matter what you do, the first two of the exercises that gives the same answer no matter what you do.* | Acute 6 |
|  |  | *The feedback is the same all the time… I feel as if when it tells me I'm lifting my thigh, I don't think it is.* | Acute 8 |
| Progress  Presentation | References relating to the graphical interfaces presented within the system. | *Sometimes on one of the exercises ,it looked as if I had hardly done any, but I knew I had done 15. So, I just…but I mean some would go up to 60 or 55 or 60 or something and one of them 20 and I was going how come that!* | Post-Acute 3 |
|  |  | *but I didn’t really know what the scorings were, didn’t study it… Ok that wasn’t clear to me.* | Post-Acute 5 |
|  |  | *Yeah the progress every time, you could see and it mostly seemed to be recorded and it was great because in the beginning you’d see very little you know and now but the end of it I am kind of scoring… That wasn’t clear initially because it is reporting on a 24 hour period.* | Acute 4 |
|  |  | *On the graph I don't know what the interpretation is supposed to be.* | Acute 6 |
| Bugs / Glitches | References relating to any technical errors that participants reported during use. | *And then it started doing really weird things in the last couple of days, because even though it registers the 15 heel slides, it then says, unexpected behaviour try again.* | Post-Acute 4 |
|  |  | *This morning it told me that an expected behaviour has occurred. I don’t know and then it did it again.. very few times have I been able to do that exercise when it’s actually registered it properly.* | Post-Acute 5 |
|  |  | *One other day something came up that said, unexpected behaviour or something like that.* | Post-Acute 1 |
|  |  | *I had just the one where it says unusual behaviour, unexpected behaviour, please repeat the exercise. I think I was saying to you that on two occasions I actually repeated the exercise… I just said there was a glitch and it didn't really bother me.* | Acute 5 |

PRIMARY THEME: USER EXPERIENCE

| Sub-theme | Description | Quotations | Participant |
| --- | --- | --- | --- |
| Overall Use | References relating to participants’ general experiences of using the system. | *I think it set me on a routine very, very quickly and a routine that I actually got to enjoy in a certain way. It was not like holding a sheet of paper… you became involved in it and so user friendly that I really think it was a great aid to me.* | Acute 5 |
|  |  | *I got quite use to it, I'm actually going to miss it. I will yeah… but just it kind of focused you a little bit more on doing them… And it does feel good then when it does say that you did them properly.* | Post-Acute 4 |
|  |  | *The first day you are quite unwell I was not very well, I had been a little bit sick at the hospital. And that was like a teddy bear, I had nurse with me and I quite liked it. The technology helped me to look at the exercise… So at first it was very interesting for me to see the exercise done, but after 3 days I knew.* | Acute 1 |
|  |  | *I think it was easy, I think it's extremely relevant and I can really see people using it big time. If I had that I would use it every time right from the very beginning. I think it's excellent.* | Acute 5 |
|  |  | *It's very helpful, it’s much better than leafing through static illustrations. It's 3D real time. It made what are otherwise boring exercises more interesting.* | Acute 9 |
|  |  | *It made it more interesting, just to have something to play with while you do the exercises.* | Acute 8 |
|  |  | *I'm going to miss the machine, I really am. It was a great help, it helped me get through it, it gave me a progress report. I knew what I was doing. It was speed, it was easy access and it was very helpful.* | Acute 9 |
| Repetition Counter | References relating to the participants’ experiences due to the repetition counter usability issues. | *I have to do it till it hurts before that will register.* | Post-Acute 2 |
|  |  | *I found it frustrating that it was meant to count and it didn't count.* | Acute 8 |
|  |  | *No and it doesn’t start straight away counting, so I do it a second time, because I still don’t know whether I'm hitting it properly.* | Acute 7 |
|  |  | *The green number, Mad erratic… I'd be more concerned from you filtering all the information, did you see oh he did twenty there and I didn't do twenty, I did the fifteen.* | Acute 5 |
| Classification / Bugs | References relating to the participants’ experiences due to the classification and technical usability issues. | *It’s just very frustrating as I say it’s on the three that are really painful to do, and you struggle through them and you think well I think I have done them fairly well and then it says unexplained behaviour do them again and you just can’t.* | Post-Acute 4 |
|  |  | *Feedback is useful and it is a motivation to try and get it harder, but there is a danger of that, in some of them I think ah it must be just the machine, because I'm sure I'm doing it right, and then you meet a physio and the physio says no you are not doing it right! and that’s very useful.* | Post-Acute 5 |
|  |  | *It just says correct. Now I know they’re not correct but from the apps perspective I am doing a knee bend. It’s not very good but I am doing a knee bend.* | Acute 2 |
|  |  | *The best part was when it told me I had done them right, and I moved on. Occasionally on the leg raised one, it told me I had lifted my leg slightly, but I didn’t feel as if I had, but asked me to redo it, so I redid it each time.* | Post-Acute 3 |
|  |  | *Yes it’s just rather infuriating, I know it’s an accurate representation that my leg isn’t getting straight enough, but it’s just a little bit disheartening when you have tried as hard as you possibly can to get the leg straight and it tells you.* | Acute 4 |

PRIMARY THEME: PERCEIVED IMPACT

| Sub-theme | Description | Quotations | Participant |
| --- | --- | --- | --- |
| Adherence | References relating to the perceived impact participants feel use of the system has on adherence to exercise. | *It kept me doing physio when I might not have done it at home, especially with various things that have been happening at home. So it kept me doing physio and made sure I did it every day.* | Post-Acute 3 |
|  |  | *Well I can 100% tell you that I had a previous knee operation and I didn’t have an app and I did the exercises as diligently and frequently as I could but I certainly didn’t do them with the thoroughness and regularity that I’ve done them this time* | Acute 3 |
|  |  | *Yeah it’s good, keeps you doing it anyway.* | Post-Acute 1 |
|  |  | *Time is short in the mornings, you might be inclined to cut short your exercise and I’ll do more of them later, but when you are using the app you stick with it.* | Post-Acute 5 |
|  |  | *I wouldn’t have done the exercises to the same degree… I just feel I possibly wouldn’t have done them as rigid as I have tried to do them. Now I have obviously missed an odd one but I mean I have tried to do them as rigidly all the time* | Acute 2 |
|  |  | *It does make you do it more and I do think it makes you try that bit harder* | Acute 6 |
|  |  | *I've been very diligent with doing the exercises and I do find it a great help.* | Acute 9 |
| Monitoring | References relating to the perceived impact participants felt that monitoring of home exercises offered. | *I found it, I have to say, it made do the exercises when I didn’t really want to do them, knowing I was being monitored, and, but I do think it helped me a lot* | Acute 2 |
|  |  | *I kind of felt that that app now was going to first of all make me do my exercises, the three times a day, and secondly at least it was recording it and you could see, that’s what I liked, you could see the progress from I’m sure you have the record there of the beginning ones weren’t great.* | Acute 4 |
|  |  | *It is useful in the fact that it definitely makes you do the exercises because you know you are being monitored. It makes them a little more interesting as well.* | Acute 8 |
|  |  | *The other thing that was good was to go in and look at your progress as well.* | Post-Acute 4 |
|  |  | *If you know someone is looking at you, I'm definitely going to do a better job of doing my exercises the right way… I was definitely much more inclined to do the exercises, if I know he is able to see.* | Post-Acute 5 |
|  |  | *I called it Big Brother, but it was watching me and no matter what I did my three lots of exercises with all the repetitions, exactly as I’ve been shown in the hospital, because the app was there monitoring and it became a little bit of a challenge for me to try and improve the scores each day that I did the app. So essential, it was an essential and hugely beneficial part of my recuperation.* | Acute 3 |
|  |  | *I definitely know, like the fact that somebody was watching it definitely made me do it.* | Acute 4 |
|  |  | *I liked to see the progress at the end of the day, or even after the session. I checked that every time to see if it has gone from red to yellow etc.* | Acute 8 |
|  |  | *I liked that, it was like a reward graph.* | Acute 9 |
| Motivation | References relating to participants’ views how the use of the system impacted their motivation to exercise. | *I enjoyed using it, it’s easy use and does motivate me to do this.* | Post-Acute 5 |
|  |  | *Oh yes it made a huge difference, that was a great motivation… It just meant that I was in control of my situation and I didn't feel the days were endless. I was looking forward to doing it to see how well I was doing.* | Acute 7 |
|  |  | *A huge incentive and even if you try it and say, I'm too tired tonight and then you say no, no I have to do it and I have to try and improve on it.* | Acute 6 |
|  |  | *The way it is is very motivational.* | Acute 3 |
|  |  | *What's the word for it, motivates it. It certainly does. It's better than a kick in the ass.* | Acute 6 |
| Classification | References relating to the perceived impact from receiving exercise technique based biofeedback. | *So certainly it gives me incentive to try and think about the exercise and getting it done properly.* | Post-Acute 5 |
|  |  | *That’s the important bit about it that it tells you straight away, you need feedback, there is no point in having the app if it doesn’t give you feedback… Because if you are waiting for someone to come in and check it out for you, that’s two to three weeks, but it’s two to three weeks of doing it wrong.* | Post-Acute 2 |
|  |  | *it’s ideal for somebody that’s coming straight out of hospital and they have to do the exercises on their own, because if you are not doing it correctly why are you bothering doing them in the first place. So that as a tool in itself is worth a hell of a lot.* | Post-Acute 3 |
|  |  | *Just the fact that it counted the repetitions and it told you whether you were doing them right or wrong. Now I know I said I didn’t like it saying that I didn’t do them correctly but it was good to know that you weren’t actually doing them correctly.* | Post-Acute 4 |
|  |  | *Yes, so the knee bend, it was telling me my knee was falling to the side, I thought actually now, now I'm doing it slightly different and I'm conscious of it and it says it’s ok* | Post-Acute 5 |
|  |  | *The app helped me because I know I was doing the exercises and I was doing them right and going ok.* | Post-Acute 5 |
| Negative Experience | References relating to participants who reported negative impact & experience in system use. | *Yes first couple of days that was very interesting and I liked the idea of looking at the little cartoon. But after 2 or 3 days it was for me, I thought it was unnecessary.* | Acute 1 |
| Improved Rehabilitation Process | References relating to participants’ views on the use of the system in rehabilitation. | *I think the fact that I was almost keen for the next session, it led to a regularity and I think that has paid huge dividends in the exercises and in the result of the exercises on the leg. I really think it was extremely beneficial.* | Acute 5 |
|  |  | *I thought it was excellent because although you go to pre-op and you get lots of information, you have quick meetings with the surgeon, everything is always in a bit of a tizzy because you are out of your comfort zone. So the information on the app, I read every single part of it, there’s a video there with a number of surgeons talking about the operation and what to expect. It’s actually very good.* | Acute 3 |
|  |  | *It was so positive. It was just brilliant. It just meant that I was in control whereas normally I would be coming home and in the hospital it would be altogether different because there's so much support there, but when you come home its gone except that I had that.* | Acute 7 |
|  |  | *I found it very helpful, I found it a fantastic aid because it was a bit like having an instructor or a tutor with me while I was doing them.* | Acute 9 |
|  |  | *Its great and it will give people confidence that even when they know that's there that having the operation in itself, knowing that there's something on after it that it will help people.* | Acute 7 |

PRIMARY THEME: REFINEMENTS

| Sub-theme | Description | Quotations | Participant |
| --- | --- | --- | --- |
| Additional Exercises | References relating to the desire for an increased library of exercises. | *I think if you gave me progression on the exercises, well done, try this one now, I’d like that.* | Post-Acute 5 |
|  |  | *I think if it was only the same exercises and I would feel sure yes I can do them, I would feel probably not as inclined to do them but if there was kind of like ok like we’ll say you have unlocked the next level, you know, now you can do this, now move on to, but I think there would have to be a moving on to the next phase to make you keep doing it.* | Acute 4 |
|  |  | *So I suppose if there was some way of making it more difficult that there was a basic set and if it could recognise that you were able to do those well and them move you onto something else perhaps.* | Post-Acute 1 |
|  |  | *Offer me more comprehensive ones than those ones, so those ones I found reasonably easy to do, so I’d like it to do the one like kneeling and leaning back, more difficult exercises.* | Post-Acute 3 |
|  |  | *Obviously I'm doing the exercise, they are much more easy for doing them, that’s probably that’s all, I'm doing quite well and it’s…it seems to be easy. Actually they are so easy that I'm nearly ready to do a little bit more… I wonder if there would be a little button saying week 1, week 2, week 3, week 1 you do that higher, week 3 you do instead of 15 you do 30 of them, these type of things would be interesting.* | Acute 1 |
|  |  | *The only thing I would say is it is only four exercises… if there was any way you could throw in the other three… Or any other different one, to add to it* | Acute 2 |
|  |  | *I think maybe another one or two exercises just to get a better variety. There's a lot of bed exercises and the ones sitting out on the bed. I think one or two added outside of the bed would really aid and abet the situation.* | Acute 5 |
| Joint Angle | References relating to the wish for a joint angle feature for knee motion in the system. | *If it was possible on the knee bend one, if it could tell you about how much you bend your knee. And I wonder, I don’t know if this is the whole…maybe it’s a bit annoying but if it would tell you when you are actually doing it.* | Post-Acute 5 |
|  |  | *The other one thing that I’d love to be able to see is you know when you do the knee bends, I’d love to be able to see what angle you got… Now it just you know then the way at least you could say well I’ve gone from a 50 to a 75 rather than just ok it looks like I am doing it ok.* | Acute 4 |
|  |  | *This is probably not possible, but to get the angle of that knee bend, if you knew that…for me that is where I'm really stuck so just to know that…I know it counts it and it said you did it right, but I'm not sure what the angle of the bend is, and I'm rather obsessed with that.* | Post-Acute 4 |
|  |  | *Is a little bit more information maybe to say you could be doing better on the knee bend… that’s the goal isn’t it to get it to a certain range like 90 or 100, that’s what it’s all about.* | Acute 2 |
|  |  | *So just something that was able to say you are not quite getting the 90 degree when you pull your leg up.* | Acute 3 |
|  |  | *Whereas if you have more information on that as to how they're progressing, you're up to 65 bend or whatever it might be you’re doing, if there was a bit more information on the progress, that would be beneficial.* | Acute 6 |
| Progress Presentation | References relating to options for improvement of the progress graphs and section presentation. | *And you know the way your Fitbit would have the circle that you have to fill the circle and obviously this system is basic bar charts… Yes that (the Fitbit) does make more sense.* | Post-Acute 3 |
|  |  | *The one thing that would be of great benefit is if there was a progressive graph not just a sort of overall, or even the graph as it is, is not explained too well. If there was an explanation of the graph as it is, that would be great.* | Acute 6 |
|  |  | *I think you know on the health app, it shows you the steps and any simple bar chart you are using. Just what does that mean.* | Post-Acute 5 |
|  |  | *If the patient is just feeling that he can see, really all the patient needs to see is a little graph going upwards.* | Acute 3 |
| Interface Graphics | References relating to refinement opportunities in the graphics of the interface and avatar. | *I suppose it’s nice to see it really but she needs to undergo a great makeover… Just a bit more human the graphics… I mean even if it was more a cartoon figure or something it doesn’t really matter.* | Post-Acute 4 |
|  |  | *I think it’s nice and clear, good if you had men and women I suppose, it would probably be good for older person if you get an older person.* | Post-Acute 5 |
|  |  | *...you have the older guy and then suddenly you have the young kid with a fancy hairdo and ripped jeans. That seems a bit...she's not right with her ripped jeans. If she's old and she's wearing ripped jeans she's certainly not right. You want somebody in they're 50s maybe.* | Acute 6 |
|  |  | *There are some disproportions of the girl on the bed, she looked very young and should probably be older.* | Acute 9 |
| Quality Score & Feedback | References relating to the opportunity to further develop the biofeedback offered. | *The feedback on the messaging I think needs improving… my first fifteen leg lifts for instance, they bear no resemblance to the fifteen I did today. You still, at the time you're doing them, you're straining with such effort that you want the recognition that you did them, you don't want a negative message and I know this is very, very difficult, how you get that message across, look we know you're trying here and you haven't quite succeeded in doing anything if you like as opposed to your last one where you get a grand correct, you made fifteen returns, so there's a bit gap between those two I think.* | Acute 5 |
|  |  | *It would be very difficult to rate it from the previous time, there is no linkage from the previous repetitions… So in a way you don’t know if you are doing better today than you did yesterday… The quality of how I’m doing them.* | Acute 2 |
|  |  | *So if somehow you were able to have something to do with the quality of the , the quality of, if you had a mark for yes having done your fifteen repetitions, on each of the exercises.* | Acute 3 |
|  |  | *Well I would like to be able to know say I did day one I did seven well and eight badly.* | Acute 4 |
|  |  | *I think the negative it could be worded a bit better, something like ok you have done the exercise, there is room for improvement… the way you would on a one to one basis, you said to me, ok you did this, in fact when we were doing the leg lifts the other day and you said, just bring it above your toe line. You didn’t say to me it’s no good if you don’t reach your toe line. That type of thing.* | Acute 5 |
|  |  | *You're doing well, you've got to do better… I don't want it to lie to me either and say, you're doing great when I know I'm not.* | Acute 6 |
| Customisation & Gamification | References relating to serious gaming aspects of customisation and gamification. | *If there was a games element to it you know you have unlocked the next level… or a medal or something.* | Acute 4 |
|  |  | *I think that'd be a mistake because then you are going to end up turning it into playtime rather than exercise time. I think it'll lose the point of it. I don’t think you should be able to manipulate too much unless it is information gathering or correcting the programme. For me this is a medical instruction for exercise to improve your health... you'd end up wasting time and not doing the exercise.* | Acute 9 |
|  |  | *Choice of male or female theme throughout the system would be nice.* | Acute 8 |
